# Fifty years of Shannon information theory in assessing the accuracy and agreement of diagnostic tests

**DOI:** 10.1007/s11517-021-02494-9

**Published:** 2022-02-23

**Authors:** Alberto Casagrande, Francesco Fabris, Rossano Girometti

**Affiliations:** 1 Dipartimento di Matematica e Geoscienze, Università degli Studi di Trieste, Trieste, Italy; 2 Istituto di Radiologia, Dipartimento di Area Medica, Università degli Studi di Udine, Ospedale S. Maria della Misericordia, Udine, Italy

**Keywords:** Diagnostic information, Diagnostic test performance, Quality measures, Inter-rater agreement, Shannon information theory, Informational diagnostic channels

## Abstract

Since 1948, Shannon theoretic methods for modeling information have found a wide range of applications in several areas where information plays a key role, which goes well beyond the original scopes for which they have been conceived, namely data compression and error correction over a noisy channel. Among other uses, these methods have been applied in the broad field of medical diagnostics since the 1970s, to quantify diagnostic information, to evaluate diagnostic test performance, but also to be used as technical tools in image processing and registration. This review illustrates the main contributions in assessing the accuracy of diagnostic tests and the agreement between raters, focusing on diagnostic test performance measurements and paired agreement evaluation. This work also presents a recent unified, coherent, and hopefully, final information-theoretical approach to deal with the flows of information involved among the patient, the diagnostic test performed to appraise the state of disease, and the raters who are checking the test results. The approach is assessed by considering two case studies: the first one is related to evaluating extra-prostatic cancers; the second concerns the quality of rapid tests for COVID-19 detection.

## Introduction

Claude Shannon’s “A mathematical theory of communication” [67], published in 1948, is the milestone paper of the new information age, flourished in the second half of 1900s, which is characterized by a finely branched network that connects each computer, smartphone, terminal, or device we use in our daily life. Shannon’s work, which describes the fundamental laws of data compression and error correction over a noisy channel, marks the birth of a unifying theory, i.e., *Information Theory* (*IT*), with profound intersections with probability, statistics, computer science, and many other fields [75]. Shannon’s paper also introduced some novel concepts which aimed to measure the quantity of information either stored in a string or transferred through a communication process: they are, respectively, (Shannon) *entropy*, related to the data compression problem, and *channel capacity*, which models the flow of information over a noisy channel, and is defined in terms of the *Mutual Information* (*MI*) between input and output.

Communication theory was the first discipline to adopt these information measures, but several attempts were made to export these concepts also in many other fields, such as statistical mechanics [38], statistical inference [48, 81], linguistics [76], taxonomy [47], psychology [8], molecular dynamics [34], computational biology [17], molecular biology [27], genomics [44], neurobiology [26], pattern recognition [49], machine learning [37], deep learning [40], computer vision [66], perception [22], image processing [30], and many others. In some cases, however, these measures have been used in an uncritical way and outside a proper theory-safe environment, so that only questionable and partial results were produced. In other cases, when the problems of these external disciplines have been interpreted in agreement with the *IT* spirit, an informational-theoretic approach to the discipline was set.

This also occurred in *medical diagnostics*, where clinical tests are performed to determine which disease or condition better explains patient’s symptoms and signs, such as the test to measure the *prostate-specific antigen* (PSA) level [63], or the genetic test to identify *cystic fibrosis* [41], or cellular analysis to detect cell-based diseases such *sickle anemia* [3], or tests based on medical imaging to ascertain or rule out the presence of breast cancer [59].

Entropy and mutual information have been successfully used many times in medical diagnostics. For example, Richman et al. [64] introduced *sample entropy* (SampEn) as a method to estimate the entropy of a system represented by a time series, and the technique has been used with success in further researches [1, 21, 54]. In [29] Faes and Porta illustrated a framework to quantify the dynamics of information in coupled physiological systems based on the notion of *conditional entropy* (CondEn); this method has been used in the neural and cardiovascular time series framework [60, 61]. In [80], Xiong et al. presented a systematic study on the performance, bias, and limitations of three entropy-based measures, to be applied in the context of dynamical systems described by real-world time series, including non-stationarities and long-range correlations. More recently *Wiener-Granger causality* (WGC) [36, 78], where a variable *X* Granger causes a variable *Y* if the information in the past of *X* improves the prediction of *Y*, was used to analyze time series. Here, *IT* methods play an important role in the definition of many of the time domain model-free measures of causality [28, 62, 69].

Another relevant application area for *IT* techniques is medical imaging registration, classification, segmentation and features extraction. Maes et al. [50] reviewed the breakthrough impact of the mutual information maximization criterion in the analysis of multispectral and multitemporal images, where proper image alignment is required to compare corresponding regions in each image volume. Uthoff and Sieren [74] used feature selection methods to quantitatively gauge intensity, texture, and shape of breast lesions; the method is based on three information measures, derived from mutual information, and combined to assess the added benefit of including a feature into the classifying set. A comprehensive review of various image segmentation techniques, also including entropy-derived measures, is given in [18].

Focusing on accuracy measured for diagnostic tests and the agreement between raters, we have to note that the disease is a hidden and objective status of the patient and the physician makes assumptions on it by interpreting the result of the test. Thus, the test is a means to extract *information* from the patient to diagnose the disease. Therefore, the most accurate diagnostic test will be the one that can extract as much information as possible: the more knowledge flows from the disease to the reader, the more accurate the diagnostic test. The information is implied at the beginning of the diagnostic process and plays a fundamental role.

In this context, physicians have to cope with two primary goals: the first one is to appraise the *Diagnostic Test Accuracy* (DTA) of a considered test. Such an evaluation also enables clinicians to identify the most effective diagnostic test among a set of possible choices, for instance, comparing digital versus film mammography in diagnosing breast cancer [59]. The second one is to establish an *Agreement Measure* (AM) to compare evaluations of the same diagnostic outcome produced by different raters or validate new rating systems or devices. For the sake of example, the agreement between ultrasound and automated breast volume scanner can be used to assess breast cancer findings [33].

DTA for dichotomous diagnostic tests has historically been based on the evaluation of sensitivity (*SE*), specificity (*SP*), as well as their derived measures, such as *likelihood ratios* [31, 77]. The main drawback of this approach is that it does not offer a single statistical measure that can summarize the global quality of a dichotomous diagnostic test [58]. The multi-valued case has been handled by selecting a different threshold for *SP* and then evaluating the *area under the curve* (AUC) of *SE* in a *Receiver Operating Characteristic* (*ROC*) analysis. In this case too, it is not clear how to compare different tests when they have the same AUC, but a different shape of the *ROC* curve or what is the best threshold to come back to the dichotomous case, when, for example, a multi-valued ranking scale is used, such as the *Breast Imaging-Reporting and Data System* (BI-RADS) or the *Prostate Imaging-Reporting and Data System* (PI-RADS).

As for the agreement measures, many different techniques have been introduced so far, but Cohen’s *κ* (kappa) [19] is undoubtedly the most popular agreement method between two raters and proved its effectiveness in the last sixty years. Nonetheless, this method suffers from some severe issues: namely, its value is strongly dependent on the prevalence of the disease [68].

Apart from the effectiveness of the methods introduced in the literature to tackle DTA and AM, all the techniques used in practice miss the information’s strategic and operative involvement. Since the final purpose of carrying out a diagnostic test is to gain information about the patient’s condition, this seems to be a significant shortcoming.

This manuscript is aimed at reviewing the last half-century of information theory in assessing the accuracy of diagnostic tests and the agreement between raters, focusing on diagnostic test performance measurements and paired agreement evaluation, presenting all the advancements in the field up to the most recent ones. We, first of all, introduce some of the central notions in Shannon’s information theory together with the medical diagnostic setting in Section 2. Then, we consider the information measures introduced to gauge the accuracy of diagnostic tests (Section 3) and those meant to evaluate the inter-rater agreement (Section 4). Section 5 discusses a recent unified approach to both quantify the accuracy of diagnostic tests and, alternatively, assess the agreement between two raters in both dichotomic and multi-valued cases. The effectiveness of this approach is tested in Section 6 by means of two clinical case studies: the first one deals with three raters of a diagnostic test to detect extra-prostatic cancers; the second is related to rapid tests for COVID-19 detection. Finally, Section 7 presents some final remarks and indicates future developments for the topic.

## Information theory and medical diagnostics

From the theoretical point of view, the correct approach to handle information measures is referring to *Shannon’s Information Theory* (IT) [67], which constitutes the mathematical apparatus underlying all current telecommunication systems, based on a rigorous and quantifiable notion of information, over which we obtain some information-derived measures, such as *entropy*, *mutual information* (*MI*) and *informational divergence* (ID). It is worth noting that Shannon entropy, based on the logarithmic function, has been proved by Khinchin [42] to be the sole measure of information that satisfies some reasonable postulates necessary to define an information measure [2] in a coherent setting.

Before discussing the results presented in medical diagnostics literature during the last fifty years, let us introduce the main actors of Shannon IT, starting with the informational divergence [45], described for the first time a few years after Shannon’s work [67]. It has the merit of being the mathematical root over which we can deduce mutual information and entropy in a natural way.

### Informational divergence, mutual information and entropy

Let *P* = {*p*_1_,*p*_2_,…,*p*_*K*_} and *Q* = {*q*_1_,*q*_2_,…,*q*_*K*_} be probability distributions; then

1 $$ \mathcal{D}({P}//{Q}) \stackrel{\textrm{\tiny{}def}}{=} {\sum}_{i=1}^{K} p_{i} \log \frac{p_{i}}{q_{i}}.  $$

is the *informational divergence* (ID), or *Kullback-Leibler divergence*, between the two PDs. ID is always greater than or equal to 0 and the strict equality holds if and only if *P* ≡ *Q* [20]. Since $\mathcal {D}({P}//{Q})=0$ iff *P* ≡ *Q*, the divergence can be interpreted as an asymmetric *pseudo-distance* among probability distributions; it is not a distance because it lacks symmetry and the triangular inequality does not hold in general [20].

Let us now consider the probability distributions *P*_*X*_ and *P*_*Y*_, associated with the random variables *X* and *Y*, and the corresponding joint probability distribution *P*_*XY*_. Then the ID

2 $$ \mathcal{D}({{P}_{\scriptscriptstyle{XY}}}//{{P}_{\scriptscriptstyle{X}} {P}_{\scriptscriptstyle{Y}}}) = {\sum}_{x,y}^{}p(x,y) \log \frac{p(x,y)}{p(x)p(y)} \stackrel{\textrm{\tiny{}def}}{=} I(X, Y) $$

can be interpreted as the (oriented) distance from the condition of independence, since *P*_*XY*_ ≡ *P*_*X*_*P*_*Y*_ implies ID equal to 0. The quantity *I*(*X*,*Y* ) is the *mutual information* between the random variables *X* and *Y*. It is symmetric (*I*(*X*,*Y* ) = *I*(*Y*,*X*)), and always non-negative, as it is a special kind of informational divergence. So we can interpret *MI* as a measure of stochastic dependence between two random variables. From the informational point of view, if *I*(*X*,*Y* ) = 0 then *X* and *Y* do not exchange information; on the contrary, if *MI* is greater than 0, it measures the quantity of information exchanged between the two random variables.

If *Y* = *X* we obtain

3 $$ \begin{array}{@{}rcl@{}} I(X, X) &=& {\sum}_{i,j}^{}p(x_{i},x_{j}) \log \frac{p(x_{i},x_{j})}{p(x_{i})p(x_{j})} \stackrel{\textrm{\tiny{}def}}{=} H(X)\\ &=& - {\sum}_{i=1}^{K} p(x_{i}) \log p(x_{i}) \geq 0 \end{array} $$

and the quantity defined on the right is the famous *Shannon entropyH*(*X*), which expresses the expected value of the random variable $\mathcal {I}(X) = -\log \text {Pr}\{X\}$, which is the *self-information* [20]. Entropy is the average quantity of information associated with a random variable, and it is simple to verify [20] that

4 $$ \begin{array}{@{}rcl@{}} &0 \leq H(X) \leq \log K \qquad &(=0 \quad \text{iff}  {P}_{\scriptscriptstyle{X}}  \text{is degenerative} ) \\ & &(=\log K \quad \text{iff} {P}_{\scriptscriptstyle{X}} \text{is uniform}) \end{array} $$

5 $$ \begin{array}{@{}rcl@{}} I(X, Y) &= H(X) - H(X/Y) = H(Y) - H(Y/X) \geq 0  \\ I(X,Y) &\leq \min \{H(X), H(Y)\} \end{array} $$

### The medical diagnostics setting

We can now translate the definitions just seen in terms useful for applications in the field of medical diagnostics. We have a state of *disease*, or pathologic state for a patient, which is described by the random variable *D*; it takes its value in the set $\mathfrak {D}=\{d_{1},d_{2},\ldots ,d_{K}\}$. Similarly, we have a random variable *R* which represents the *report*; it is the outcome of a diagnostic test, which is interpreted by a clinician we call *rater*. *R* takes its value in the set $\mathfrak {R}=\{r_{1},r_{2},\ldots ,r_{M}\}$. We usually assume there are only two mutually exclusive states *D* of *disease* for a patient (*K* = 2), either the disease is *present* (*D* = 1) or *absent* (*D* = 0) [58, 83]; *p*_*D*_(1) = *p*(*D* = 1) and *p*_*D*_(0) = *p*(*D* = 0) are the corresponding probabilities. As for the report *R*, we can have several cases. The first one is the *dichotomous* case, in which there are only two kinds of responses: *R* = 1 indicates the presence of the disease, and we call it *positive*; *R* = 0 indicates the absence of the disease, and we call it *negative*; *p*_*R*_(1) = *p*(*R* = 1) and *p*_*R*_(0) = *p*(*R* = 0) are the corresponding probabilities. Another important case is the *multi-valued* one, as in the *breast imaging-reporting and data system* (BI-RADS) report, where we can set a 5-point malignancy scale: 1 = negative; 2 = benign; 3 = probably benign; 4 = suspicious; 5 = highly suspicious. The last case is that in which the quantity describing the output of the test is continuous, which is the *continuous* case.

If we restrict our attention to the dichotomous case, the four possible combinations of the considered diagnostic test outcome together with the standard of reference result can be represented by a 2 × 2 table known as *confusion matrix*, which contains the number of *true positives* (*TP*), *true negatives* (*TN*), *false positives* (*FP*) and *false negatives* (*FN*) reports. By using these quantities, we can define the following measures:

6 $$ \begin{array}{ccc} \textit{SE} \stackrel{\textrm{\tiny{}def}}{=} p(R=1/D=1) = \frac{TP}{TP+FN} &\hfill & \textit{FNR} \stackrel{\textrm{\tiny{}def}}{=} p(R=0/D=1) = \frac{FN}{TP+FN} \\ \text{Sensitivity} & & \text{False negative rate} \\ \textit{FPR} \stackrel{\textrm{\tiny{}def}}{=} p(R=1/D=0) = \frac{FP}{FP+TN} & & \textit{SP} \stackrel{\textrm{\tiny{}def}}{=} p(R=0/D=0) = \frac{TN}{FP+TN} \\ \text{False positive rate} & & \text{Specificity} \end{array} $$

where *p*(*r*/*d*) = *p*(*R* = *r*/*D* = *d*) is the conditional probability that the report *R* is *r*, given that the disease variable *D* equals *d*. Regardless of the patient condition, the diagnosis provided by the diagnostic test is either *R* = 1 or *R* = 0. Thus, *p*(*R* = 1/*D* = *d*) + *p*(*R* = 0/*D* = *d*) = 1 for all the conditions *d* ∈{0,1}. The value *p*(*D* = 1) is the *pre-test* probability of the disease, while *p*(*D* = 1/*R* = *r*) is the *post-test* probability of the disease when the outcome of the diagnostic test is *r* ∈{0,1}.

## Diagnostic information measures and test accuracy

During the last fifty years, the literature about Shannon information theory in clinical diagnostics has been mainly devoted to gauging the quantity of diagnostic information extracted from clinical tests in specific medical fields.

The first contribution that applied IT techniques to dealing with medical diagnostics seems to be a paper by Good and Card dated 1971 [35]. That work was aimed at maximizing the expected utility of a diagnostic process, where the utility considers both the patient’s condition and the various costs associated with the diagnostic process. Since utilities are generally difficult to estimate, the authors suggested some substitutes for them, called *quasi-utilities*, and they also identified as possible candidates the informational divergence (there called *dinegentropy*), the mutual information (there called *mean information transfer*), and the *expected weight of evidence*. This paper has the merit of introducing, for the first time, “the concept of the patient and doctor as forming a communication or information channel.” The authors used the ID $\mathcal {D}({\boldsymbol {q}}//{\boldsymbol {\pi }})$ to evaluate the stochastic distance between ***q*** = {*q*_1_,*q*_2_,…,*q*_*m*_}, which is an estimation, during a diagnostic process, of the probabilities *m* mutually exclusive diseases *d*_1_,*d*_2_,…,*d*_*m*_, and the initial estimates ***π*** of the same probabilities, or the vector of initial probabilities. The physician has to estimate the vector ***q*** “by means of tests, calculations and judgements.” The authors themselves also suggested choosing the test that maximizes $\mathcal {D}({\boldsymbol {q}}//{\boldsymbol {\pi }})$. Later, they unraveled the connection with the Shannon communication channel and showed that maximizing $\mathcal {D}({\boldsymbol {q}}//{\boldsymbol {\pi }})$ “comes to the same thing as the maximization of the mean information transfer” [35, page 181]. In the authors’ language, this is equivalent to the mutual information *I*(***D***,***F***/*K*), where *K* is a conditional variable corresponding to the knowledge of the physician, ***D*** is the probability distribution of the diseases *p*(*d*_*i*_) = *π*_*i*_, and ***F*** is a vector associated with the chosen test. So, *I*(***D***,***F***/*K*) has to be maximized by the physician “by choice of the test.” Moreover, since *MI* is the information transmission rate and the expected weight of evidence is the logarithm of a likelihood ratio, the measures are related to the quantity of information extracted by the diagnostic test in both cases.

After this first contribution, there have been several other attempts to introduce Shannon-like methods in medical diagnostics. Another important attempt was the one suggested by Metz, Goodenough, and Rossmann [52], who in 1973 used the mutual information in conjunction with *ROC* curves. The authors proposed to gauge the imaging system performance by using *ROC* curve data and, successively, to evaluate radiographic images. This approach relates each point of the *ROC* curve as 1-*SP* varies with the mutual information *I*(*D*,*R*) between the random variables *D*, which represents the two states of disease of the patient, and *R*, which corresponds to the diagnostic report of the reader. This method can be used in two ways: the first one quantifies the maximum amounts of information available on two different *ROC* curves to compare the quality of the two systems used to generate the curves themselves. The second way measures the quantity of information obtained by a rater operating at any two points of the same *ROC* curve or a single point on two *ROC* curves; in this case, the authors measure the information extracted in a diagnostic process by using mutual information. This approach is similar to that of Good and Card [35] even though [52] did not cite it.

In 1978 Okada [56] used *MI* and a custom-tailored weighted entropy for a slightly different goal: reducing the amount of clinical data by eliminating relatively insignificant items.

The paper [52] by Metz, Goodenough, and Rossman has been a source of inspiration for many subsequent works in several areas of clinical diagnostics. For example, Diamond et al. used the mutual information *I*(*D*,*R*) to gauge the diagnostic effectiveness of different test combinations in the clinical diagnosis of coronary artery disease [24]. The method was compared with an alternative approach which evalutates the average value of the difference between the probability of the disease before and after testing, i.e., Δ*p* = |*p*(*d*/*r*) − *p*(*d*)|. An essential contribution of this paper is that of recognizing the dependency of mutual information from the prevalence of disease; the issue was solved in the Appendix by integrating *MI* for all the prevalences to obtain an average value to be used for coronary angiography. The subsequent work of Diamond et al. [25] used mutual information to evaluate the information content of the electrocardiographic ST-segment response to exercise relative to the diagnosis of angiographic coronary artery disease.

In paper [65] by Rifkin, the author analyzed the increase of information available for a diagnostic test when one increases the number of outcomes associated with the test results.

Somoza and Mossman [55, 70–73] instead investigated how to choose the best cutoff in diagnostic tests with a continuous response characterized by a *ROC* curve. Their approach, which extended [52], selected the best cutoff by maximizing the mutual information of the diagnostic test on the *ROC* curve. The authors used this technique to evaluate the measure of Rapid Eye Movement (R.E.M.) latency as a diagnostic test for depression [70].

In 1990, Asch et al. [6] criticized the use of mutual information, as introduced in [52]; they stated that *MI* is not able to correctly detect the “prognostic information” that results from the application of a clinical test. They provided an example in which they try to evaluate the information conveyed by a positive test result as a consequence of a change from *p*(*D* = 1) = 0.1 of the pre-test probability of disease to *p*(*D* = 1/*R* = 1) = 0.9 of the post-test probability of disease, knowing that we have obtained a positive test result. They evaluated this information by computing the difference *H*(*D*) − *H*(*D*/*R* = 1), which corresponds to an unweighted sub-component of ordinary mutual information. Since the a priori and a posteriori probabilities are complementary, they obtained a difference equal to 0. They imputed this to a flaw of information theory since “patients are not indifferent to a chance of disease of *q* and a chance of 1 − *q*.” They proposed, as an alternative, the use of the difference *p*(*D* = 1/*R* = 1) − *p*(*D* = 1), already discussed in [24], but not mentioned in its bibliography. With abuse of language, they called this approach “Linear information theory” although it deviates from classical Shannon information theory, it wasn’t supported by any formal framework, and the word “information,” which in their paper denoted a difference between probabilities, was not properly used. The contribution was harshly criticized by Diamond which stated that the example they provided does “not represent a failure of the theory; it represents a failure to appreciate what the theory is about” [23]. This commentary had a reply [7] with a dispute about the concept of “average change of probability” Δ*p*.

The issue of evaluating the information conveyed by a diagnostic test once a test result is obtained was repurposed by Benish several years later [9]. He suggested replacing the old formula *H*(*D*) − *H*(*D*/*R* = 1), criticized in [6], with the informational divergence $\mathcal {D}({(D/R=1)}//{D})$, which solves the pitfall discussed in that paper; it corresponds to measuring the stochastic distance between the post-test and the pre-test probability of disease, knowing that we have obtained a positive test result. The author stressed that it “is not a measure of the absolute amount of information that a test provides.” The ID function was proposed in the same year by Lee [46] to select diagnostic tests to rule in or rule out a disease; in this context, the authors suggested the evaluation of $\mathcal {D}({(R/D=1)}//{(R/D=0))})$ or $\mathcal {D}({(R/D=0)}//{(R/D=1))})$.

The “absolute amount of information that a test provides,” cited in the paper [9] by Benish, has been discussed in two subsequent contributions by the same author [10, 11], where it is shown that *I*(*D*,*R*) “quantifies the expected value of the amount of information the test provides.” Even though this concept is not new at all — e.g., we cited [24, 35, 52] as prior examples– one has to admit that [11] is the first paper that extensively and systematically described, discussed, and put into a correct environment the problem of measuring the information carried out by a diagnostic test. In this contribution, we can also find a quotation of the channel capacity, defined as the maximization of *MI* across all possible distributions of the pre-test probability of disease; here *MI* is also used as an index of the performance of a diagnostic test, as the title itself suggests. The use of *I*(*D*,*R*) as the correct tool to manage medical diagnostics information has been supported even from an axiomatic perspective in another paper by Benish [12].

Several other contributions followed these seminal papers, focusing on the practical application of IT methods to actual clinical cases, especially mutual information. For example, in [57] the topic studied is the problem of quantifying the performances of two tests for major depressive disorder, which are the dexamethasone suppression test (DST) and the thyroid-stimulating hormone test (TSH). In contrast, in [4] the authors need to evaluate the effectiveness of GDx nerve fiber analyzer parameters in the diagnosis of glaucoma. In another work [79] the *MI* is used to extract the most informative mammographic features for breast cancer diagnosis, while the authors of [5] use *MI* to detect occlusal caries lesions.

Recently, some authors have further developed the old idea — advocated by Good and Card [35] and successively taken up by several other authors — of simply measuring the bare amount of information flowing from the disease to the physician through the use of a diagnostic test. This approach is interesting from the theoretical perspective, but it does not offer the physician an operative tool to compare two diagnostic tests whose accuracy is usually specified through sensitivity and specificity. Moreover, the physician would need a method to measure the global test accuracy using a single number. The first one to pursue this goal was Benish [13], who applied the information theory concept of channel capacity to diagnostic test performance, deriving an expression for channel capacity in terms of test sensitivity and specificity, and finding the prevalence of disease that allows this maximization. It is worth noting that Benish has been the most contributor, during the last 20 years, in the field of application of Shannon information theory to medical diagnostics [9–14].

**Fig. 1:**
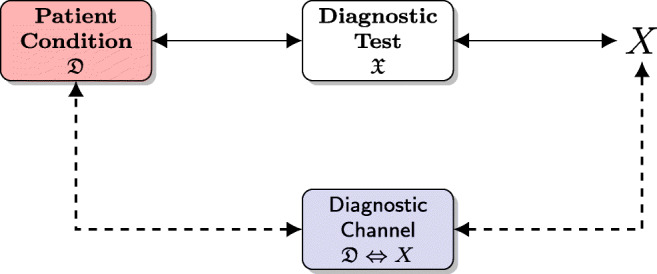
The *diagnostic channel* connects the patient disease $\mathfrak {D}$ with the outcome (random variable) *X* of the diagnostic test interpreted by the rater $\mathfrak {X}$; it is formed by the chain patient condition $\mathfrak {D}$ ⇔ diagnostic test performed by $\mathfrak {X} \Leftrightarrow X$, and it is briefly indicated as $\mathfrak {D}\Leftrightarrow X$

Subsequently, Girometti and Fabris [32] independently developed an IT framework for diagnostic test accuracy by defining a *diagnostic channel* that connects the patient disease $\mathfrak {D}$ with the outcome *X* of the diagnostic test interpreted by the rater $\mathfrak {X}$ (see Fig. 1). While this idea is not new, it properly contextualizes *MI* usage and formalizes the notion of the diagnostic channel.

The same paper also introduced a normalized measure of the test performance in the interval [0,1] — based on *MI* as a function of sensitivity and specificity — called the *information ratio* (IR) of the diagnostic test, which expresses a global measure of the test accuracy and is independent form the prevalence of the disease. Since prevalence is an important variable that can dramatically change the quantity of information measured by the test, and then the quality of the same test, it has been proposed to integrate *MI* over all the prevalences (e.g., see [24, Appendix]) and normalizing the Area Under the Curve (AUC) with respect to the maximum area available for the standard of reference. A similar method is also discussed for the case of multi-valued diagnostic tests with a variable threshold such as BI-RADS. Section 5 will present this approach.

## Informational inter-rater agreement

The literature about IT methods to evaluate paired agreement is much more limited. To our best knowledge, Klemens was the first one to measure agreement by applying a Shannon-like method. He used a normalized weighted *MI* as an index of inter-rater agreement [43] and, for each couple of readings *i*,*j*, the weights *w*_*ij*_ were such that *w*_*ij*_ = 1 if *i* = *j*, and *w*_*ij*_ = 0 if *i*≠*j*. This approach is equivalent to taking into account only the cases in which the raters completely agree and decoupling the agreement component of *MI* from the disagreement part. He then normalized this skewed *MI* with respect to the sum of the entropies *H*(*X*) and *H*(*Y* ). The paper by Kang et al. [39] uses instead *MI* to quantify the information shared between outcomes of multiple healthcare surveys. However, this approach dissected *MI* among the agreement and the disagreement components, too, and it distorted the spirit and the axiomatics of the Shannon’s *MI* function, which averages all the components.

**Fig. 2:**
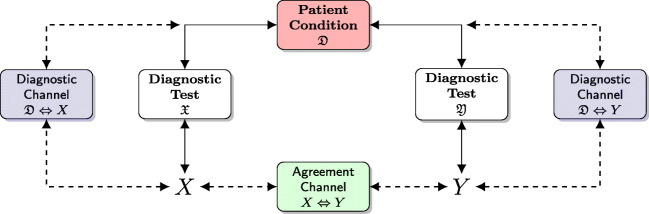
The *diagnostic channel* connects the patient disease $\mathfrak {D}$ with the outcome (random variable) *X* (*Y* ) of the diagnostic test interpreted by the rater $\mathfrak {X}$ ($\mathfrak {Y}$); it is formed by the chain patient condition $\mathfrak {D}$ ⇔ diagnostic test performed by $\mathfrak {X} \Leftrightarrow X$, and it is briefly indicated as $\mathfrak {D}\Leftrightarrow X$. The *agreement channel* connects the random variables *X* and *Y*, that express the raters outcomes. They are the terminals of the chain *X* ⇔ diagnostic test performed by $\mathfrak {X}$ ⇔ patient condition $\mathfrak {D} \Leftrightarrow $ diagnostic test performed by $\mathfrak {Y} \Leftrightarrow Y$. It is briefly indicated as *X* ⇔ *Y*

Only recently, Casagrande et al. [15] proposed the use of the classical Shannon orthodox approach also in the agreement context (see also [16]). This is done by introducing an *agreement channel*, which connects *X* and *Y* as the terminals of the chain *X*⇔ diagnostic test performed by $\mathfrak {X}$ ⇔ patient condition $\mathfrak {D}$ ⇔ diagnostic test performed by $\mathfrak {Y}$ ⇔ *Y*, which corresponds to the concatenation of the two diagnostic channels $X\Leftrightarrow \mathfrak {D}$ and $\mathfrak {D}\Leftrightarrow Y$ (see Fig. 2); we briefly indicate the agreement channel as *X* ⇔ *Y*, and it constitutes the framework to evaluate AM using the so-called *informational agreement* (*IA*), which is a normalized measure in the interval [0,1], that can directly be compared with Cohen’s *κ*.

## An IT-based unifying approach

In this section, we recall the main elements associated with the model we need to measure the performance of a diagnostic test and the agreement between two raters. The starting point is the definition of a diagnostic channel and an agreement channel.

### Measuring the quality of a diagnostic test

Based on the literature of the last fifty years, we can now state that mutual information has definitely been accepted as the correct method to measure the quantity of information extracted by a diagnostic test [11–13, 24, 32, 35, 52, 73]. Concerning the medical diagnostics setting Section 2.2, the information exchanged between *D* and *R* is measured by

7 $$ I(D,R)= {\sum}_{\stackrel{\scriptstyle {d \in \mathfrak{D}}} {r \in \mathfrak{R}}}^{} p(d,r)\log_{2}\frac{p(d,r)}{p(d)p(r)}  $$

with the logarithm taken to the base 2. Using the Bayes rule *p*(*d*,*r*) = *p*(*d*)*p*(*r*/*d*), which is $p(r)={\sum }_{d \in \mathfrak {D}}^{}p(d,r)={\sum }_{d \in \mathfrak {D}}^{}p(d)p(r/d)$, we have

8 $$ \begin{array}{@{}rcl@{}} I(D,R) &=& {\sum}_{\stackrel{\scriptstyle {d \in \mathfrak{D}}} {r \in \mathfrak{R}}}^{} p(d)p(r/d)\log_{2}\frac{p(r/d)}{p(r)}\\ &=& {\sum}_{\stackrel{\scriptstyle {d \in \mathfrak{D}}} {\scriptstyle {r \in \mathfrak{R}}}}^{} p(d)p(r/d)\log_{2}\frac{p(r/d)}{{\sum}_{d^{\prime} \in \mathfrak{D}}^{}p(d^{\prime})p(r/d^{\prime})} \end{array} $$

In the dichotomous case, the prevalence of the disease *P*_*D*_=def*p*(*D* = 1) equals 1 − *p*(*D* = 0), because *p*(*d*) is a probability. Hence, $p(r)={\sum }_{d^{\prime } \in \mathfrak {D}}^{}p(d^{\prime })p(r/d^{\prime })$ can be rephrased as *p*(*r*/*D* = 0) + *p*(*D* = 1)(*p*(*r*/*D* = 1) − *p*(*r*/*D* = 0)). Moreover, *p*(*r* = 1/*d*) = 1 − *p*(*r* = 0/*d*) for any *d*, thus, due to *SE* and *SP* definitions in terms of *p*(*r*/*d*) (see Eq. 6), the mutual information between *D* and *R* equals

9 $$  \begin{array}{llll} I(D,R)= & P_D (\log_{2}{(1-\textit{SE})} + \textit{SE} (\log_{2}{\textit{SE}} - \log_{2}{(1-\textit{SE})}))\\ & + (1-P_D) (\log_{2}{(1-\textit{SP})} + \textit{SP} (\log_{2}{\textit{SP}} - \log_{2}{(1-\textit{SP})}))\\ & -((1-\textit{SP}) + P_D (\textit{SE} + \textit{SP} - 1)) \log_{2} [(1-\textit{SP})\\ &+ P_D (\textit{SE} + \textit{SP} - 1)]-(\textit{SP} + P_D (1 - (\textit{SE} + \textit{SP}))) \log_{2} [\textit{SP}\\ &+ P_D (1 - (\textit{SE} + \textit{SP}))]. \end{array} $$

Equation 9 proves that the mutual information between the rater and the disease exclusively depends on *SE*, *SP*, and *P*_*D*_. On the one hand, this measure is subject to the prevalence, which is not always known and may be biased; on the other hand, once *SE* and *SP* are measured, we can evaluate the mutual information itself for any possible prevalence by using Eq. 9. In order to stress this last aspect, we may refer to the mutual information between *D* and *R* — i.e., *I*(*D*,*R*) — also as *MI*_*SE*,*SP*_(*P*_*D*_) or, whenever both *SE* and *SP* can be deduced from the context, as *MI*(*P*_*D*_).

In order to define a prevalence-independent metric for rater performances, we can account for all the possible mutual information values for any prevalence, which is done by integrating *MI* over all the prevalences of disease in the interval [0,1] [24, 32]

10 $$ \overline{\textit{MI}} \stackrel{\textrm{\tiny{}def}}{=} {{\int}_{0}^{1}} \textit{MI}(P_D)  \mathrm{d} P_D  $$

**Fig. 3:**
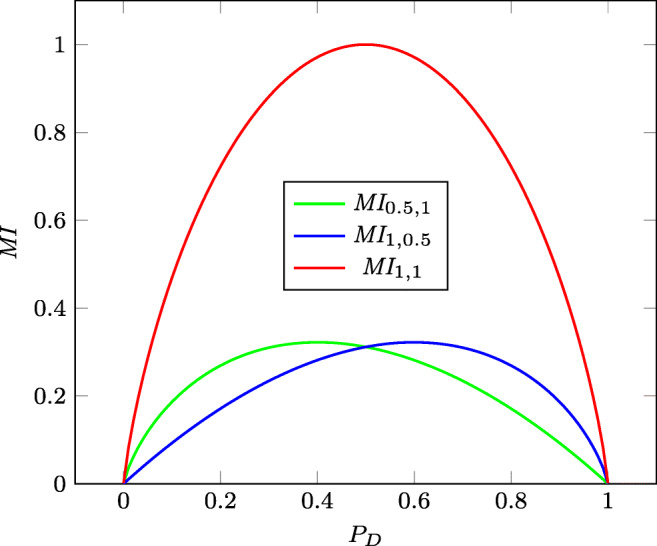
*MI*_0.5,1_ (*SE* = 0.5 and *SP* = 1), *MI*_1,0.5_ (*SE* = 1 and *SP* = 0.5), and *MI*_1,1_ (*SE* = 1 and *SP* = 1) as the prevalence of the disease varies in [0,1]

This corresponds to evaluating the AUC of the *MI* curve over all the prevalences. The *MI* curve associated with the case *SE* = 1 and *SP* = 1 is the one having the greatest admissible AUC. This *MI* curve is the *standard of reference* (SR) and its AUC equals $\overline {\textit {MI}}_{1,1}=1/\ln 4$. Figure 3 depicts the standard of reference *MI*_1,1_ together with the curves *MI*_0.5,1_ (*SE* = 0.5 and *SP* = 1) and *MI*_1,0.5_ (*SE* = 1 and *SP* = 0.5) as the prevalence of the disease varies in the closed interval [0,1]. Note that when the curves intersect, as in the example of Fig. 3, one can specify the interval with the best behavior for each curve. In this case, we have the green curve better for *P*_*D*_ < 0.5 and the blue one for *P*_*D*_ > 0.5.

The *information ratio* *IR* [32] is $\overline {\textit {MI}{}}$ normalized with respect to the maximum value of it, i.e., $\overline {\textit {MI}{}}_{1,1}$,

11 $$ \textit{IR} \stackrel{\textrm{\tiny{}def}}{=} \frac{\overline{\textit{MI}{}}}{\overline{\textit{MI}{}}_{1,1}} = \ln 4 {{\int}_{0}^{1}} \textit{MI}(P_D)  \mathrm{d} P_D. $$

It is worth to notice that the value of *IR* still depends on both *SE* and *SP*.

**Fig. 4:**
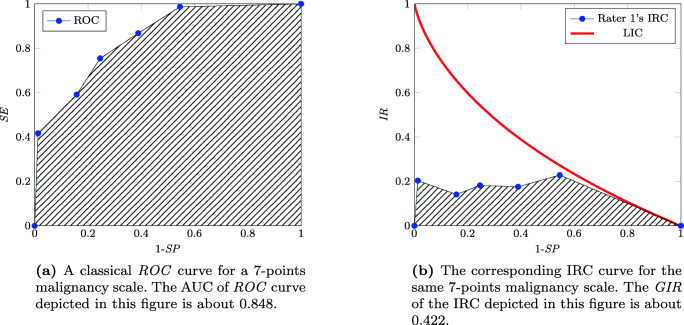
Two purely illustrative *ROC* and *GIR* curves for a 7-point malignancy scale

As far as the multi-valued case is concerned, we can refer again to [32], where the global quality of the test is evaluated by changing the threshold of *SP*, so as to obtain an *IR* value for each value of 1 −*SP*. In Fig. 4a we can see an example of a classical *ROC* curve for a 7-point BI-RADS test. The corresponding *information ratio curve* IRC is shown in Fig. 4b; we have an *IR* value for each threshold 1 −*SP* and the AUC of the curve is related with the *limit information curve* LIC, drawn by fixing *SE* = 1 for all values of 1 −*SP*, which corresponds to the curve associated with the maximum amount of information we can gain for each value of 1 −*SP*. The AUC of the LIC curve is computed in [32] and is equal to 2 − *π*^2^/6 ≈ 0.35506. The normalization of the IRC’s AUC with respect to the AUC of the LIC curve gives the *global information ratio* *GIR*

12 $$ \textit{GIR} \stackrel{\textrm{\tiny{}def}}{=} \frac{AUC_{IRC}}{2- \pi^{2}/6}  $$

which expresses a global, prevalence-independent, normalized evaluation of the quality of a multi-valued diagnostic test. It can be thought of as the information counterpart of a ROC curve.

### Measuring the agreement between raters

The problem of evaluating the agreement between two raters is solved by using the approach depicted in [15], which consists in expressing it as the quantity of information flowing through the *agreement channel* of Fig. 2, which is the virtual channel connecting the random variables *X* and *Y* through the information path *X*⇒ rating by $\mathfrak {X}$ ⇒ condition $\mathfrak {D}$ ⇒ rating by $\mathfrak {Y}$ ⇒ *Y*. It is so because *I*(*X*,*Y* ) is a measure of the stochastic dependence between *X* and *Y*. Since $I(X,Y) \leq {\min \limits } \{H(X), H(Y)\}$ (see Eq. 5), we can normalize *I*(*X*,*Y* ) with respect to ${\min \limits } \{H(X), H(Y)\}$; this leads to the *informational agreement* *IA*

13 $$ \textit{IA}(X,Y) \stackrel{\textrm{\tiny{}def}}{=} \frac{I(X,Y)}{\min \{H(X), H(Y)\}} $$

whose value ranges in the interval [0,1]. As pointed out in [15], contrary to what happens with Cohen’s *κ*, *IA* correctly measures the stochastic distance between *P*_*XY*_ and *P*_*X*_*P*_*Y*_, which is the distance of the two raters from the condition of independence. This means that *IA* gauges the (normalized) amount of information exchanged between the two raters. Furthermore, this measure can be used in both the dichotomic and multi-valued scale ratings.

## Two case studies from clinical diagnostics

To assess the global quality of the approaches depicted in Section 5, which is to measure the quality of the readings of some raters that have to evaluate the outcomes of a diagnostic test and the mutual agreement between a couple of raters, we considered two case studies; the first one deals with raters of a diagnostic test to detect extra-prostatic cancers; the second one is related to COVID-19 rapid detection.

### Detecting extra-prostatic cancers

For this analysis, we took the original data set used with the paper [82]. In this study, investigators assessed whether Magnetic Resonance Imaging (MRI) of the prostate added value to clinical models in diagnosing so-called pathological stage ≥T3 prostate cancer, i.e., cancer with extra-prostatic extension into surrounding soft tissue and invasion of the seminal vesicles at pathological analysis after surgery. Preoperative knowledge of stage ≥T3 is essential to both plan the type of surgery — for instance, to plan whether to perform nerve-sparing surgery– and predict the risk of recurrent prostate cancer after primary treatment.

In the source study, three different radiologists with 8, 6, and 2 years of experience in prostate MRI (raters $\mathcal {R}_1$, $\mathcal {R}_2$ and $\mathcal {R}_3$, respectively) prospectively evaluated MRI examinations performed to stage prostate cancer before radical prostatectomy. They attributed an MRI stage on a rank scale (T1c, T2a, T2b, T2c, T3a, and T3b). On this basis, we have performed three kinds of analysis. The first one was devoted to testing the diagnostic accuracy of each radiologist in assessing pathological stage ≥T3 under the form of the *IR*. In order to achieve this goal, MRI readings were dichotomized by assuming that the MRI stage ≥T3a was the cutoff for the pathological stage ≥T3 diagnosis.

In the second analysis, we evaluated readers’ accuracy in diagnosing pathological stage ≥T3 on a multi-valued basis, i.e., by obtaining the *GIR* and *ROC* curves built upon all the rank values attributed by radiologists in image analysis.

Lastly, we focused on assessing pairwise inter-rater agreement — i.e., $\mathcal {R}_1$ vs $\mathcal {R}_2$, $\mathcal {R}_1$ vs $\mathcal {R}_3$, and $\mathcal {R}_2$ vs $\mathcal {R}_3$. This has been done by computing the information agreement for both dichotomic and multi-valued cases and Cohen’s *κ* for the dichotomic case alone.

#### Results for extra-prostatic cancers

**Fig. 5:**
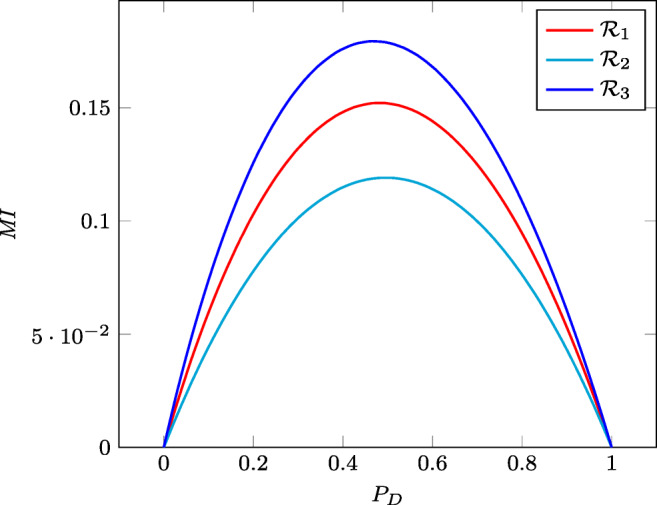
The mutual information of each of the extra-prostatic cancer raters $\mathcal {R}_1$, $\mathcal {R}_2$, and $\mathcal {R}_3$ against the standard of reference as the prevalence of the disease varies in the interval [0,1]

Table 1 shows the *IR* of the three raters with respect to the standard of reference for the data set associated with the search of extra-prostatic cancers; it also contains the parameters usually computed to determine the quality of a diagnostic test, which are essentially sensitivity, specificity, false positive and false negative rates. Figure 5 shows instead the variation of *MI* with respect to the prevalence of the disease.

**Table 1:** The sensitivity, specificity, false positive, false negative rates, and the value of *IR* for each of the extra-prostatic cancer raters $\mathcal {R}_1$, $\mathcal {R}_2$, and $\mathcal {R}_3$ with respect to the standard of reference

	$\mathbf {\mathcal {R}_1}$	$\mathbf {\mathcal {R}_2}$	$\mathbf {\mathcal {R}_3}$
*SE*	0.62	0.67	0.58
*SP*	0.82	0.73	0.88
*FNR*	0.38	0.33	0.42
*FPR*	0.18	0.27	0.12
*IR*	0.142	0.112	0.167

**Fig. 6:**
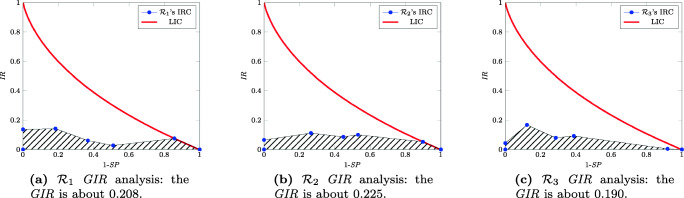
GIR analysis of the three extra-prostatic cancer raters versus the standard of reference

Figure 6 contains the *IR* profile while changing the *SP* threshold, so as to generate the *GIR* curve and the corresponding normalized value. The third point starting from the left corresponds to the standard cutoff 1 − 4|5 − 6 — to be intended as surgery is required from rate 5 on — we can appreciate that in all the three cases it has the maximum value of *IR*; this means that the threshold used is the best possible since it carries the maximum amount of diagnostic information.

**Fig. 7:**
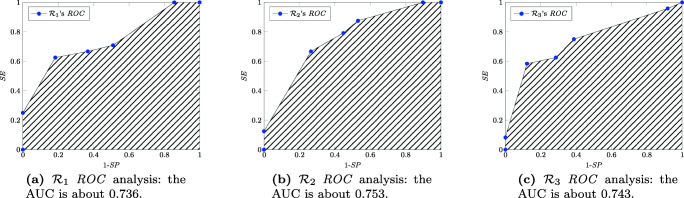
ROC analysis of the three extra-prostatic cancer raters versus the standard of reference

The results of the standard *ROC* analysis are reported in Fig. 7. It is not clear how to validate the best threshold for the *ROC* curve.

As for the agreement comparison, the results are available in Table 2; we have evaluated the *IA* for the dichotomous and the multi-valued cases and Cohen’s *κ* for the dichotomous case alone.

#### Discussion on extra-prostatic cancers

Table 1 shows that the *IR* s of $\mathcal {R}_1$, $\mathcal {R}_2$, and $\mathcal {R}_3$ are 0.142, 0.112, and 0.167, respectively. Since they lay within a narrow range, the three raters are almost equivalent, with $\mathcal {R}_3$ performing a little better than the others and $\mathcal {R}_1$ is the second best. Figure 5 shows that this ordering holds for all the prevalences, because the *MI* curve related to $\mathcal {R}_3$ is always above those of the other two raters, while that associated to $\mathcal {R}_2$ is below the curve of $\mathcal {R}_1$ for all the possible prevalences.

The *IR* s of all the raters are quite small in absolute terms with respect to the theoretical maximum values for *IR*, i.e., 1. While this is partially due to both the low sensitivity (0.62, 0.67, and 0.58, respectively), which is typical for this kind of measure, and the not so high specificity (0.82, 0.73, and 0.88, respectively) of the raters, this drift is quite frequent in the general case for informational measures that, being based on entropy, are able to discriminate even modest changes in rater performances when both sensitivity and specificity are close to 1.

Figure 6 shows the *GIR* diagrams of the three raters together with the corresponding values — i.e., 0.208, 0.225, and 0.190 for $\mathcal {R}_1$, $\mathcal {R}_2$, and $\mathcal {R}_3$, respectively. Also, in this case, we can consider the quality of the raters almost equivalent, but with a different ordering as $\mathcal {R}_2$ performs better than the other two and $\mathcal {R}_1$ follows. It is worth noticing that this last ordering seems to be more tuned with the one subtended by the experience of the three raters — i.e., $\mathcal {R}_1$ in first place, $\mathcal {R}_2$ in second, and $\mathcal {R}_3$ in third. This seems to suggest that the *GIR* analysis, based on a variation of the threshold for specificity, is more coherent than the simple *IR* analysis based on a fixed threshold.

The AUC under the *ROC* curves of Fig. 7 offers a slightly different and less convincing vision of the raters’ performance, since in this case the most experienced rater $\mathcal {R}_1$, having 8 years of experience in prostate MRI, is considered the worst rater with $\mathcal {R}_2$ topping the other two.

The agreement analysis, interestingly, offers support to the idea that $\mathcal {R}_2$ and $\mathcal {R}_3$ are the furthest away, as specified in *IR* and *GIR* analysis, since for all three methods used, (dichotomous *IA*, multi-valued *IA* and Cohen’s *κ*, see Table 2) it comes out that $\mathcal {R}_2$ vs $\mathcal {R}_3$ shows the worst value of the agreement. Since the *IA* for the multi-valued scale is by far the most refined method from the theoretical point of view, we can accept the fact that $\mathcal {R}_1$ vs $\mathcal {R}_2$ have the best agreement, also because the other two methods, dichotomous *IA* and *κ*, would suggest that the best agreement is between $\mathcal {R}_1$ and $\mathcal {R}_3$, which seems not coherent with the scale of years of experience of the raters.

**Table 2:** Agreement between each pair of extra-prostatic cancer raters $\mathcal {R}_1$ vs $\mathcal {R}_2$, $\mathcal {R}_1$ vs $\mathcal {R}_3$, and $\mathcal {R}_2$ vs $\mathcal {R}_3$, both in the dichotomous and in the multi-valued case, expressed by the *IA*. The last column contains Cohen’s *κ* values

**Raters**	***IA*** *dichotomous*	***IA*** *multi-valued*	**Cohen’s** **κ**
$\mathcal {R}_1$ vs $\mathcal {R}_2$	0.259	0.361	0.558
$\mathcal {R}_1$ vs $\mathcal {R}_3$	0.490	0.337	0.741
$\mathcal {R}_2$ vs $\mathcal {R}_3$	0.222	0.263	0.487

In conclusion, we could suggest that $\mathcal {R}_2$ is the best rater among the three, $\mathcal {R}_1$ comes in second place, and $\mathcal {R}_3$ is by far the worst of the three. In this sense the *GIR* and the multi-valued *IA* appear to be the best tools to use when evaluating the quality of a reader or the agreement between readers, at least when we have a multi-valued scale of ratings.

### Evaluating the effectiveness of serology tests for COVID-19 detection

A possible application for the analysis described in Section 5.1 is the comparison of the accuracy of COVID-19 tests. For the sake of example, we considered the data reported in [53] and we analyzed the comparison between RT-PCR, which is the standard of reference for COVID-19 diagnosis, and two automated and one rapid lateral flow immunoassays for the detection of anti-SARS-CoV-2 antibodies. These essays highlight SARS-CoV-2 specific antibodies in blood samples and allow rapid identification of the COVID-19 disease in the considered subjects. We limited our analysis to the Euroimmun Anti-SARS-CoV-2 ELISA IgG and IgA combined assays (Euroimmun, Luebeck, Germany), the Maglumi™ 2019-n-Cov IgG and IgM combined immunoassays (CLIA), and the 2019-n-CoV IgG/IgM combined rapid test cassette (LaboOn Time) (LabOn Time, Bio Marketing Diagnostics, or Akiva, Israel).

By using the number of true positives, RT-PCR positive, true negative, and negative RT-PCR of the considered combined tests, that are reported in [53, Table 1], we calculated the sensitivities and specificities of the assays. Then, their *IR* s were assessed and the tests were sorted according to their *IR* s to identify the most accurate test on average over all possible prevalences of disease. Furthermore, we computed the mutual information of the investigated tests and RT-PCR for the values 1/8, 2/8, …, and 7/8 of the prevalences of disease by using Eq. 9. For each of these prevalences, we re-sorted the tests according to their mutual information with respect to the standard of reference and we established the more effective tests among those analyzed for the specific value of *P*_*D*_.

#### Results of the analysis of COVID-19 tests

Table 3 shows the values of *IR* for the tests ELISA, CLIA and LaboOn Time, together with the corresponding sensitivity, specificity, false positive and false negative rates.

**Table 3:** Sensitivity, specificity, false positive, false negative rates and the value of *IR* for the Euroimmun Anti-SARS-CoV-2 ELISA IgG and IgA combined assays, Maglumi™ 2019-n-Cov IgG and IgM combined immunoassays (CLIA) and LaboOn Time kit

	**ELISA**	**CLIA**	**LaboOn Time**
*SE*	0.844	0.631	0.719
*SP*	0.875	1.000	1.000
*FNR*	0.156	0.369	0.281
*FPR*	0.125	0.000	0.000
*IR*	0.392	0.417	0.504

**Fig. 8:**
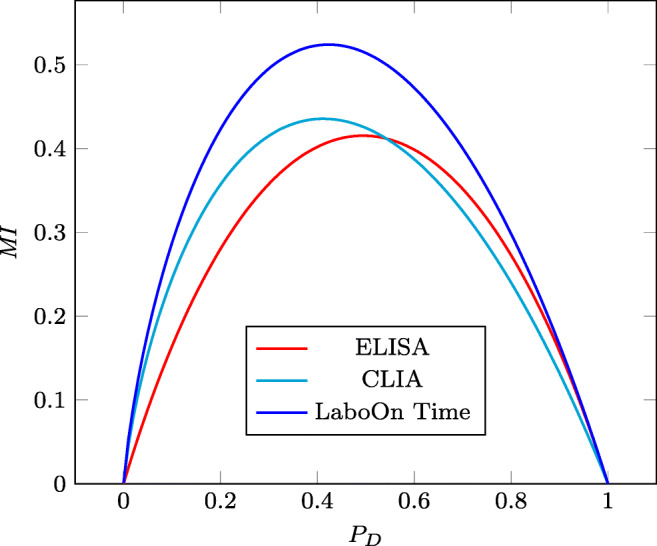
The *MI* curves of the considered COVID-19 antibodies tests as a function of the prevalence of disease

Figure 8 shows the corresponding *MI* curves as a function of the prevalence of disease. Note that the ELISA and CLIA curves intersect for *P*_*D*_ ≈ 0.55.

Table 4 reports the mutual information of the considered COVID-19 antibodies tests versus the standard of reference as the prevalences of the disease varies in {1/8,2/8,…,7/8}.

**Table 4:** The mutual information of three COVID-19 antibodies tests versus the standard of reference, i.e., RT-PCR, as the prevalences of the disease varies in the set {1/8,2/8,…,7/8} (see Section 6.2)

**Tests**	*P*_*D*_ **=** **0.125**	*P*_*D*_ **=** **0.250**	*P*_*D*_ **=** **0.375**	*P*_*D*_ **=** **0.500**	*P*_*D*_ **=** **0.625**	*P*_*D*_ **=** **0.750**	*P*_*D*_ **=** **0.875**
ELISA	0.197	0.323	0.393	0.415	0.389	0.316	0.190
CLIA	0.280	0.392	0.433	0.425	0.374	0.286	0.161
LabOn	0.329	0.465	0.519	0.514	0.457	0.353	0.202

#### Discussion on the COVID-19 tests

As far as the analysis of the COVID-19 antibodies tests is concerned, the *IR* s reported in Section 6.2.1 suggests that, if there are no preferences to the ability to identify positive cases with respect to the capability of discharging the negative ones, whenever the prevalence of the disease is unknown, LaboOn Time is preferable to the other two tests for all the values of prevalence, with CLIA in second position among the three.

Moreover, Table 4 indicates that CLIA performs better than ELISA for all the preferences in {1/8,2/8,3/8,4/8}, but the latter is more accurate than the former for the prevalences 5/8, 6/8, and 7/8. This is also visible in Fig. 8 where the mutual information curves of these two tests and RT-PCR intersect on *P*_*D*_ ≈ 0.55.

## Discussion and conclusions

Information theory has been used in many areas, such as computer science, physics, biology, linguistics, taxonomy, psychology and many others. It has been applied also in medical diagnostics, for example, to study systems represented by a time series, or to describe the dynamics of information in coupled physiological systems, or to extract features in medical imaging registration, classification and segmentation.

As for the problem of assessing the accuracy and agreement of diagnostic tests, many intriguing results have been obtained in the last fifty years. Nevertheless, even though these contributions are based on consolidated mathematical tools [67], they have not been considered for daily clinical practice, which instead keeps employing, in both DTA and AM contexts, more classical approaches. This discrepancy may be due to several reasons.

In some cases, the proposed methods merely used Shannon functions, such as entropy, mutual information, and informational divergence, as flat formulas to derive different custom-modified measures to express DTA or AM. This approach, when not supported by any axiomatic framework, led to both questionable and difficult-to-be-interpreted results.

In other cases, even though the suggested measures remained inside the orthodoxy depicted by Shannon, their advantages with respect to the mainstream statistical approaches, such as the commonly used Cohen’s *κ*, remained obscure to the vast audience of physicians partially because of the lack of the necessary software tools to broadly test and, possibly, adopt them. Perhaps, the most important motivation for not using Shannon-derived measures for DTA and AM in medical diagnostics is that they have seldom been operatively compared with the tools daily used in clinical diagnostic.

The path depicted in [15, 16, 32] tries to overcome all these limitations. Clinical tests are modeled as a channel (the diagnostic channel) that routes information about the disease from patients to their diagnosticians and, because of this, Shannon-theory can be applied to evaluate a normalized measure of the information acquired by using the tests themselves in both dichotomic and multi-valued cases [32]. Analogously, the agreement channel between pairs of raters is used to gauge the quantity of information virtually exchanged by raters themselves in their evaluations and, as a consequence, their agreement [15, 16]. The comparisons of the proposed measures against the standard dogmatic statistical tools, such as Cohen’s *κ*, Scott’s *π*, or Bangdiwala’s *B*, suggested that the former perform better than the latter in both cited tasks.

So, why are these Shannon-oriented measures still far away from being widely adopted? On the one hand, software tools that allow non-experts in information theory to evaluate these metrics are still missing; this aspect discourages physicians from using the discussed approach in their research manuscripts and standard practices and it delays the penetration of the information theory tools in the clinical community. On the other hand, the absence of any absolute qualitative reference scale for the new metrics plays a role in this lack of interest too. In other terms, no scale that establishes whether one can consider an *IR*, *GIR*, or *IA* value to be “good” or “bad” has been proposed yet. It is worth noticing that, even in the context of classical statistical tools, these scales are either missing or, in the best case, totally arbitrary and devoid of any objective foundation, such as the widespread-adopted linear scale proposed in [51] to rate Cohen’s *κ* — i.e., [0,0.2)(“*none to slight*”), [0.2,0.4) (“*fair*”), [0.4,0.6) (“*moderate*”), [0.6,0.8) (“*substantial*”), and [0.8,1.0) (“*perfect or almost perfect agreement*”).

We feel confident in foretelling that the mentioned obstacles to the adoption of the IT-based evaluation approach in the clinical domain will be removed in the next years, which will release new advances in medical diagnostics.
